# Supplementary material for: COZOID: contact zone identifier for visual analysis of protein-protein interactions
Source: BMC Bioinformatics. 2018 Apr 6;19:125. doi: 10.1186/s12859-018-2113-6 (PMC5889581; doi:10.1186/s12859-018-2113-6)
Supplement: Supplementary file 2 — Software build. Executable binary file of the software tool. (ZIP 118,784 kb) [file 12859_2018_2113_MOESM2_ESM.zip › cozoid/java/docs/beansbinding-1.2.1-doc.zip!/org/jdesktop/el/ExpressionFactory.html]

ExpressionFactory (Beans Binding)


---


|  |  |  |
| --- | --- | --- |
| **Overview** \| **Package** \| **Class** \| **Use** \| **Tree** \| **Deprecated** \| **Index** \| **Help** | |  |
| **PREV CLASS**   **NEXT CLASS** | **FRAMES**    **NO FRAMES**     **All Classes** |  |
| SUMMARY: NESTED \| FIELD \| CONSTR \| METHOD | DETAIL: FIELD \| CONSTR \| METHOD |  |


---


## org.jdesktop.el Class ExpressionFactory

```
java.lang.Object
  org.jdesktop.el.ExpressionFactory
```

**Direct Known Subclasses:**: ExpressionFactoryImpl

---

``` public abstract class ExpressionFactory extends java.lang.Object ```

Parses a `String` into a `ValueExpression` or
`MethodExpression` instance for later evaluation.

Classes that implement the EL expression language expose their
functionality via this abstract class. There is no concrete implementation
of this API available in this package. Technologies such as
JavaServer Pages and JavaServer Faces provide access to an
implementation via factory methods.

The `createValueExpression(org.jdesktop.el.ELContext, java.lang.String, java.lang.Class )` method is used to parse expressions
that evaluate to values (both l-values and r-values are supported).
The `createMethodExpression(org.jdesktop.el.ELContext, java.lang.String, java.lang.Class , java.lang.Class [])` method is used to parse expressions
that evaluate to a reference to a method on an object.

Unlike previous incarnations of this API, there is no way to parse
and evaluate an expression in one single step. The expression needs to first
be parsed, and then evaluated.

Resolution of model objects is performed at evaluation time, via the
`ELResolver` associated with the `ELContext` passed to
the `ValueExpression` or `MethodExpression`.

The ELContext object also provides access to the `FunctionMapper`
and `VariableMapper` to be used when parsing the expression.
EL function and variable mapping is performed at parse-time, and
the results are
bound to the expression. Therefore, the `ELContext`,
`FunctionMapper`,
and `VariableMapper`
are not stored for future use and do not have to be
`Serializable`.

The `createValueExpression` and
`createMethodExpression` methods must be thread-safe. That is,
multiple threads may call these methods on the same
`ExpressionFactory` object simultaneously. Implementations
should synchronize access if they depend on transient state.
Implementations should not, however, assume that only one object of
each `ExpressionFactory` type will be instantiated; global
caching should therefore be static.

The `ExpressionFactory` must be able to handle the following
types of input for the `expression` parameter:

- Single expressions using the `${}` delimiter
  (e.g. `"${employee.lastName}"`).
- Single expressions using the `#{}` delimiter
  (e.g. `"#{employee.lastName}"`).
- Literal text containing no `${}` or `#{}`
  delimiters (e.g. `"John Doe"`).
- Multiple expressions using the same delimiter (e.g.
  `"${employee.firstName}${employee.lastName}"` or
  `"#{employee.firstName}#{employee.lastName}"`).
- Mixed literal text and expressions using the same delimiter (e.g.
  `"Name: ${employee.firstName} ${employee.lastName}"`).

The following types of input are illegal and must cause an
`ELException` to be thrown:

- Multiple expressions using different delimiters (e.g.
  `"${employee.firstName}#{employee.lastName}"`).
- Mixed literal text and expressions using different delimiters(e.g.
  `"Name: ${employee.firstName} #{employee.lastName}"`).

**Since:**
:   JSP 2.1

---

**Constructor Summary**

`ExpressionFactory()`


| **Method Summary** | |
| --- | --- |
| `abstract  java.lang.Object` | `coerceToType(java.lang.Object obj, java.lang.Class<?> targetType)`             Coerces an object to a specific type according to the EL type conversion rules. |
| `abstract  MethodExpression` | `createMethodExpression(ELContext context, java.lang.String expression, java.lang.Class<?> expectedReturnType, java.lang.Class<?>[] expectedParamTypes)`             Parses an expression into a `MethodExpression` for later evaluation. |
| `abstract  ValueExpression` | `createValueExpression(ELContext context, java.lang.String expression, java.lang.Class<?> expectedType)`             Parses an expression into a `ValueExpression` for later evaluation. |
| `abstract  ValueExpression` | `createValueExpression(java.lang.Object instance, java.lang.Class<?> expectedType)`             Creates a ValueExpression that wraps an object instance. |

**Methods inherited from class java.lang.Object**

`clone, equals, finalize, getClass, hashCode, notify, notifyAll, toString, wait, wait, wait`

**Constructor Detail**

### ExpressionFactory

```
public ExpressionFactory()
```


**Method Detail**

### createValueExpression

```
public abstract ValueExpression createValueExpression(ELContext context,
                                                      java.lang.String expression,
                                                      java.lang.Class<?> expectedType)
```

:   Parses an expression into a `ValueExpression` for later
    evaluation. Use this method for expressions that refer to values.

    This method should perform syntactic validation of the expression.
    If in doing so it detects errors, it should raise an
    `ELException`.

    :   **Parameters:**: `context` - The EL context used to parse the expression. The `FunctionMapper` and `VariableMapper` stored in the ELContext are used to resolve functions and variables found in the expression. They can be `null`, in which case functions or variables are not supported for this expression. The object returned must invoke the same functions and access the same variable mappings regardless of whether the mappings in the provided `FunctionMapper` and `VariableMapper` instances change between calling `ExpressionFactory.createValueExpression()` and any method on `ValueExpression`. Note that within the EL, the ${} and #{} syntaxes are treated identically. This includes the use of VariableMapper and FunctionMapper at expression creation time. Each is invoked if not null, independent of whether the #{} or ${} syntax is used for the expression. : `expression` - The expression to parse: `expectedType` - The type the result of the expression will be coerced to after evaluation. **Returns:**: The parsed expression **Throws:**: `java.lang.NullPointerException` - Thrown if expectedType is null.: `ELException` - Thrown if there are syntactical errors in the provided expression.

---


### createValueExpression

```
public abstract ValueExpression createValueExpression(java.lang.Object instance,
                                                      java.lang.Class<?> expectedType)
```

:   Creates a ValueExpression that wraps an object instance. This
    method can be used to pass any object as a ValueExpression. The
    wrapper ValueExpression is read only, and returns the wrapped
    object via its `getValue()` method, optionally coerced.

    :   **Parameters:**: `instance` - The object instance to be wrapped.: `expectedType` - The type the result of the expression will be coerced to after evaluation. There will be no coercion if it is Object.class,

---


### createMethodExpression

```
public abstract MethodExpression createMethodExpression(ELContext context,
                                                        java.lang.String expression,
                                                        java.lang.Class<?> expectedReturnType,
                                                        java.lang.Class<?>[] expectedParamTypes)
```

:   Parses an expression into a `MethodExpression` for later
    evaluation. Use this method for expressions that refer to methods.

    If the expression is a String literal, a `MethodExpression`  is created, which when invoked, returns the String literal,
    coerced to expectedReturnType. An ELException is thrown if
    expectedReturnType is void or if the coercion of the String literal
    to the expectedReturnType yields an error (see Section "1.16 Type
    Conversion").

    This method should perform syntactic validation of the expression.
    If in doing so it detects errors, it should raise an
    `ELException`.

    :   **Parameters:**: `context` - The EL context used to parse the expression. The `FunctionMapper` and `VariableMapper` stored in the ELContext are used to resolve functions and variables found in the expression. They can be `null`, in which case functions or variables are not supported for this expression. The object returned must invoke the same functions and access the same variable mappings regardless of whether the mappings in the provided `FunctionMapper` and `VariableMapper` instances change between calling `ExpressionFactory.createMethodExpression()` and any method on `MethodExpression`. Note that within the EL, the ${} and #{} syntaxes are treated identically. This includes the use of VariableMapper and FunctionMapper at expression creation time. Each is invoked if not null, independent of whether the #{} or ${} syntax is used for the expression. : `expression` - The expression to parse: `expectedReturnType` - The expected return type for the method to be found. After evaluating the expression, the `MethodExpression` must check that the return type of the actual method matches this type. Passing in a value of `null` indicates the caller does not care what the return type is, and the check is disabled.: `expectedParamTypes` - The expected parameter types for the method to be found. Must be an array with no elements if there are no parameters expected. It is illegal to pass `null`. **Returns:**: The parsed expression **Throws:**: `ELException` - Thrown if there are syntactical errors in the provided expression.: `java.lang.NullPointerException` - if paramTypes is `null`.

---


### coerceToType

```
public abstract java.lang.Object coerceToType(java.lang.Object obj,
                                              java.lang.Class<?> targetType)
```

:   Coerces an object to a specific type according to the
    EL type conversion rules.

    An `ELException` is thrown if an error results from
    applying the conversion rules.

    :   **Parameters:**: `obj` - The object to coerce.: `targetType` - The target type for the coercion. **Throws:**: `ELException` - thrown if an error results from applying the conversion rules.


---


|  |  |  |
| --- | --- | --- |
| **Overview** \| **Package** \| **Class** \| **Use** \| **Tree** \| **Deprecated** \| **Index** \| **Help** | |  |
| **PREV CLASS**   **NEXT CLASS** | **FRAMES**    **NO FRAMES**     **All Classes** |  |
| SUMMARY: NESTED \| FIELD \| CONSTR \| METHOD | DETAIL: FIELD \| CONSTR \| METHOD |  |


---
